# Predictors of vision impairment in Multiple Sclerosis

**DOI:** 10.1371/journal.pone.0195856

**Published:** 2018-04-17

**Authors:** Bernardo Sanchez-Dalmau, Elena H. Martinez-Lapiscina, Irene Pulido-Valdeolivas, Irati Zubizarreta, Sara Llufriu, Yolanda Blanco, Nuria Sola-Valls, Maria Sepulveda, Ana Guerrero, Salut Alba, Magi Andorra, Anna Camos, Laura Sanchez-Vela, Veronica Alfonso, Albert Saiz, Pablo Villoslada

**Affiliations:** 1 Department of Ophthalmology, Hospital Clinic, Barcelona, Spain; 2 Department of Neurology and Institut d’Investigacions Biomèdiques August Pi Sunyer (IDIBAPS), University of Barcelona, Barcelona, Spain; 3 Trial Form Support, Barcelona, Spain; Bascom Palmer Eye Institute, UNITED STATES

## Abstract

Visual impairment significantly alters the quality of life of people with Multiple Sclerosis (MS). The objective of this study was to identify predictors (independent variables) of visual outcomes, and to define their relationship with neurological disability and retinal atrophy when assessed by optical coherence tomography (OCT). We performed a cross-sectional analysis of 119 consecutive patients with MS, assessing vision using high contrast visual acuity (LogMar), 2.5% and 1.25% low contrast visual acuity (Sloan charts), and color vision (Hardy-Rand-Rittler plates). Quality of vision is a patient reported outcome based on an individual's unique perception of his or her vision and was assessed with the Visual Functioning Questionnaire-25 (VFQ-25) with the 10 neuro-ophthalmologic items. MS disability was assessed using the expanded disability status scale (EDSS), the MS functional composite (MSFC) and the brief repetitive battery-neuropsychology (BRB-N). Retinal atrophy was assessed using spectral domain OCT, measuring the thickness of the peripapillar retinal nerve fiber layer (pRNFL) and the volume of the ganglion cell plus inner plexiform layer (GCIPL). The vision of patients with MS was impaired, particularly in eyes with prior optic neuritis. Retinal atrophy (pRNFL and GCIPL) was closely associated with impaired low contrast vision and color vision, whereas the volume of the GCIPL showed a trend (p = 0.092) to be associated with quality of vision. Multiple regression analysis revealed that EDSS was an explanatory variable for high contrast vision after stepwise analysis, GCIPL volume for low contrast vision, and GCIPL volume and EDSS for color vision. The explanatory variables for quality of vision were high contrast vision and color vision. In summary, quality of vision in MS depends on the impairment of high contrast visual acuity and color vision due to the disease.

## Introduction

Visual impairment is a common feature in people with multiple sclerosis (MS)[1, 2]. High contrast visual acuity (HCVA) assessment is considered not to be sufficiently sensitive to capture visual impairment in MS patients[3], and low contrast visual acuity (LCVA)[4] and color vision[5] are more sensitive parameters of vision for MS patients[2, 6, 7]. Quality of vision is a patient reported outcome based on an individual's unique perception of his or her vision. The quality of vision in MS patients can be evaluated using the validated Visual Functioning Questionnaire-25 (VFQ25)[8] with the addition of 10 neuro-ophthalmological items[9], which would help to improve the characterization of the disease impact in people with MS.

Considering the negative impact of visual impairment on MS patients[1], understanding how specific visual abnormalities or retinal atrophy impair the quality of vision will be particularly relevant to improve the care of people with MS[10]. Moreover, defining these relationships could identify the most informative visual tests to include in multi-domain disability scores to monitor MS. Therefore, the objective of this study was to investigate the contribution of retina atrophy and disability to vision, and to identify the main predictors (significant independent variables in the multivariate regression) of visual outcomes.

## Methods

### Study population

The *Barcelona MS-VisualPath cohort* is an ongoing prospective study of patients with MS conducted at the Hospital Clinic of the University of Barcelona, Spain. The design and methods employed by the MS-VisualPath cohort have been described in detail elsewhere[11]. Briefly, the cohort included MS patients diagnosed according to McDonald Criteria[12] without any psychiatric, neurological or ocular disorders that may interfere the aims of the study. We excluded measurements from eyes with severe refractive errors (myopia > −6.0 dp or an axial eye length >26 mm; hypermetropia > 5 dp; cylinder > 3 dp), optic nerve drusen, cataracts, current or prior glaucoma, acute optic neuritis (ON) (<6 months), or other causes of visual loss not attributable to MS. The first consecutive 119 patients of this prospective cohort were included in this cross-sectional analysis. Patients were given a complete examination that included neurological evaluation, visual function testing and optical coherence tomography.

### Ethical statement

The Institutional Review Board at the Hospital Clinic of Barcelona approved the study and all participants provided their written informed consent prior to enrollment.

### Clinical evaluation

We collected demographic (gender and age) and MS-related data (disease sub-type, disease duration since first symptom onset, history of prior ON, type of disease modifying treatment -DMT- and clinical disability) from the subjects, and they all underwent visual testing and OCT evaluation during remission (patients were > 30 days from relapse). The presence of prior ON was assessed in the electronic medical records, as indicated previously[13], considering classical clinical presentation semiology and standard examinations described previously[14]. Eyes classified as non-ON eyes have neither prior nor acute ON.

We assessed global neurological disability using the Expanded Disability Status Scale (EDSS)[15] and the Multiple Sclerosis Functional Composite (MSFC)[16]. Cognitive performance was evaluated with the Brief Repeatable Battery-Neuropsychology test (BRB-N), including separate scores for verbal memory, visual memory, executive function (e.g. Symbol Digit Modality Test -SDMT) and verbal fluency[17].

A trained optometrist carried out the ophthalmological evaluations separately on each eye (visual acuity and color vision). Before assessing visual acuity and color vision, refraction was evaluated in all patients, even in those wearing their own glasses or contact lens. Optical refraction was corrected with the prescription lenses necessary in each case, positive lenses for hyperopia, negative lenses for myopia or cylinder lenses for astigmatism. The trial lenses frame was used to correct refraction at the same time. The optometrist (SA) evaluated monocular and binocular high visual acuity, low visual acuity and color vision. We evaluated HCVA using ETDRS charts and transformed the scores into logMar units for statistical analysis. LCVA was scored with the 2.5% and 1.25% low-contrast Sloan charts[4] and it are based on the number of letters correctly identified (up to 70). Color vision acuity was assessed using Hardy-Rand-Rittler (HRR) plates[18], and based on the number of symbols identified correctly (up to 36)[19].

The impact of vision was evaluated with a self-reported quality of vision assessment using VFQ25 (global and with the 10 neuro-ophthalmological supplement), as described previously[20]. The cut-off of abnormal visual quality was defined as: Global score < 88; 10-neuro-ophthalmological items < 79; and Combined score < 85[9].

### Retinal image acquisition and analysis

Retinal scans were performed at a single center by a trained technician (SA) using a Spectralis® SD-OCT device (Heyex 5.30 Heidelberg Engineering, Heidelberg, Germany) in eye-tracking mode, under standard ambient lighting (80–100 foot-candelas) and without pupillary dilation. Spherical errors were adjusted prior to each measurement and the pRNFL thickness (μm) was measured using a 12-degree diameter ring scan automatically centered on the optic nerve head (100 ART; 1,536 A Scans per B scan). The macular scan protocol involved a 20x20 degree raster scan (horizontal orientation) centered on the fovea, taking 25 horizontal sections separated by 240 μm and with a mean ART≥9 [512 A Scans per B scan].

Retinal layer segmentation was performed automatically using the in-built HRA/Spectralis Viewer Module (v.5.7.5.0) for peripapillary scans and the Viewer Module (6.0c version) for macular raster-scans to quantify the macular GCIPL volume. A single optometrist (SAA) reviewed all the images from the automated segmentation and performed manual correction for obvious errors. All images were carefully reviewed to ensure they fulfilled OSCAR-IB and APOSTEL criteria [21, 22]. Conversion of volumes (original measurement in mm^3^) to thicknesses (in μM) of the different layers was conducted using the following equation:

$$\left(1)\;\text{Thickness}\;(\mu m)=(4*\text{Volume}({\mu m}^{3}))/(\pi *{\left(6000\mu m\right)}^{2}\right)$$

### Statistical analysis

Categorical variables were compared using a Chi-squared or Fisher´s exact test. Continuous variables were compared with an independent two-sample t-test after confirming their normal distribution (Shapiro-Wilks test) or with a Mann-Whitney-Wilcoxon U test when a normal distribution could not be assumed. Linear regression analyses with dummy variables (binary variables (0 or 1) for categorical variables such as sex or previous ON) were used to assess the association between the clinical outcome measures and retinal atrophy assessed on an individual patient level in a model that included age, sex, history of MS-ON, EDSS and the use of DMTs. Additionally, linear regression analyses with dummy variables were used to identify predictors (independent variables) of visual outcomes. We selected the significant variables in the univariate linear regression and they were included in the multivariate model for each visual variable. A stepwise selection method was applied to select the significant variables in the final model. Analyses were performed on a patient level in order to relate the global outcomes (quality of vision (VFQ-25 and VFQ25+10items [8, 9, 20]), disability scales) with visual acuity or retinal thickness. For the multiple regression analysis we make use of the vision variables that should be more informative for global scales (quality of vision or neurological disability (EDSS)) and for this reason we made use of the binocular visual acuity (HCVA, LCVA and HRR). We used the mean of both eyes instead of adjusting for inter-eye correlation because such approach provides very similar results as previously described[14]. All p-values were two-tailed and they were considered significant at p ≤0.05. Statistical analyses (VA) were performed using the SAS Enterprise Guide 7.1 software. Raw data of the study is available in S1 and S2 Files.

## Results

### Characteristics of the MS cohort

The cohort was composed of 119 patients with MS, two thirds of whom were female. The mean disease duration of the cohort was 12 years (SD 7.5) and the patients were predominantly of the RRMS subtype (105, 88.2%), while there were 10 SPMS (8.4%) and 4 PPMS (3.4%) patients. There were 65 patients with no prior ON, whereas 54 had suffered a previous episode of ON in at least one of their eyes. Most of patients were receiving a DMT, with 57 patients treated with interferon beta (47.8%), 15 with glatiramer acetate (12.6%), 2 with natalizumab (1.7%) and 13 with fingolimod (10.9%). There were 30 untreated patients (25.3%), which included patients with each progressive subtype. The patients were mildly disabled, with a median EDSS of 2.0 (range 1–7.5), a mean MSFC of 0.31 (SD 1.1) and a mean z score with the BRB-N of 0.19 (SD 0.8). When comparing the demographic and clinical characteristics of the cohort as a function of prior ON or not, the only significant difference beyond visual acuity, was that patients who had suffered a previous ON performed worse in the neuropsychological tests than patients without prior ON (**Table 1**).

**Table 1:** Demographic, clinical and OCT characteristics of the MS cohort. MS patients are stratified as cases with prior optic neuritis (ON) and cases without prior ON (NON) in either eye.

	MS			
	All (N = 119)	NON-MS (N = 65)	ON-MS (N = 54)	p value
**Sex M/F**^1^	37/82 (31/69)	20/45 (31/69)	17/37 (31/69)	ns
**Age**^2^	43.9 (9.5)	44.9 (9.5)	42.6 (9.4)	ns
**Disease duration**^2^	12.01 (7.5)	12.09 (7.9)	11.9 (6.9)	ns
**Subtype**^1^				ns
**RR**	105 (88.2)	54 (83.1)	51 (94.4)	
**SP**	10 (8.4)	7 (10.7)	3 (5.6)	
**PP**	4 (3.4)	4 (6.2)	0 (0)	
**DMTs**^1^				ns
**none**	30 (25.3)	21 (32.3)	9 (16.7)	
**IFNB**	57 (47.8)	28 (43.1)	29 (53.7)	
**GA**	15 (12.6)	6 (9.2)	9 (16.6)	
**NTZ**	2 (1.7)	2 (3.1)	0 (0.0)	
**FTY**	13 (10.9)	7 (10.8)	6 (11.1)	
**Other**	2 (1.7)	1 (1.5)	1 (1.8)	
**EDSS**^3^	2.0 (1.00, 7.50)	2.0 (1.00, 7.50)	1.5 (1.00, 6.50)	ns
**MSFC (Z score)**^2^	0.31 (1.1)	0.33 (1.4)	0.28 (0.5)	ns
**T25WT**	5.09 (2.1)	5.45 (2.5)	4.61 (1.3)	ns
**9HPT**^4^	21.8 (5.2)	22.2 (5.5)	21.3 (4.6)	ns
**PASAT**	46.7 (12.2)	46.2 (11.9)	47.2 (12.6)	ns
**BRB-N (Z score)**^2^	0.19 (0.8)	-0.20 (0.5)	0.64 (0.9)	0.0465
**SDMT**^2^	54.3 (12.9)	48.0 (11.2)	62.7 (10.7)	0.0442
**HCVA (LogMar)**^2^	0.00 (0.13)	-0.02 (0.09)	0.03 (0.16)	ns
**2.5%LCVA**^2^	20.4 (10.7)	21.1 (11.1)	19.5 (10.4)	ns
**1.25%LCVA**^2^	7.79 (8.3)	9.95 (9.2)	5.17 (6.2)	0.0279
**Color vision (HRR)**^2^	33.5 (4.5)	34.5 (3.3)	32.2 (5.4)	0.0448
**VFQ-25**^2^	89.5 (11.1)	89.4 (11.4)	89.5 (10.9)	ns
**VFQ-25+10items**^2^	90.6 (10.5)	90.6 (10.8)	90.6 (10.2)	ns
**pRNFL (μm)**^2^	85.7 (17.3)	95.3 (14.8)	75.7 (13.7)	<0.0001
**GCIPL**^2^				
**mm**^3^	0.95 (0.15)	1.02 (0.13)	0.84 (0.10)	0.0003
**μM**	33.59 (5.30)	36.07 (4.59)	29.70 (3.53)	

^1^number (%)

^2^mean (SD)

^3^median (min; max)

^4^dominant hand

### Visual outcomes

**Fig 1:**
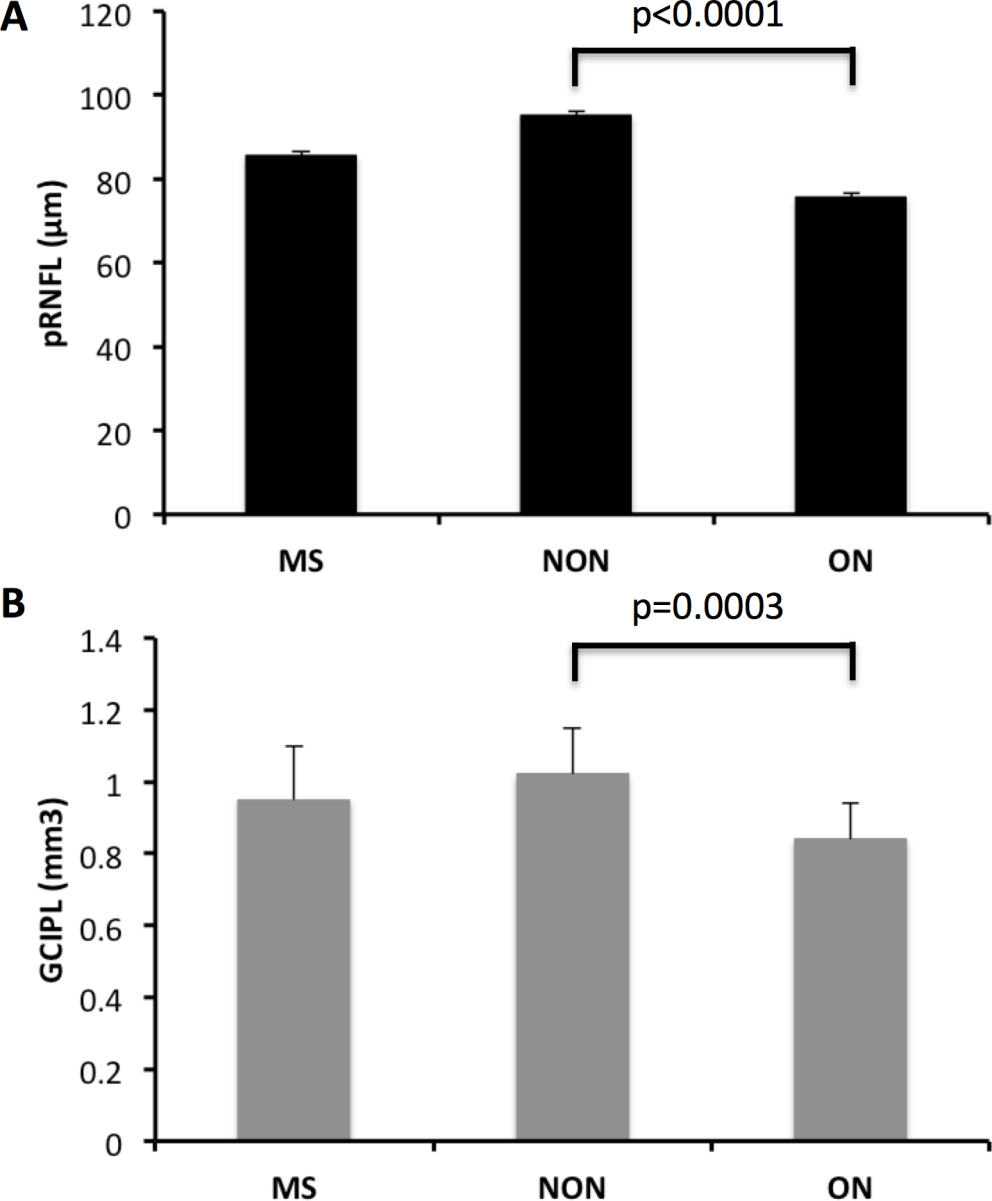
Retinal atrophy in MS assessed by OCT. The graphs shows the mean pRNFL thickness (A) or GCIPL volume (B) for the overall cohort, and for patients with prior optic neuritis (ON) or with no prior optic neuritis (NON).

**Fig 2:**
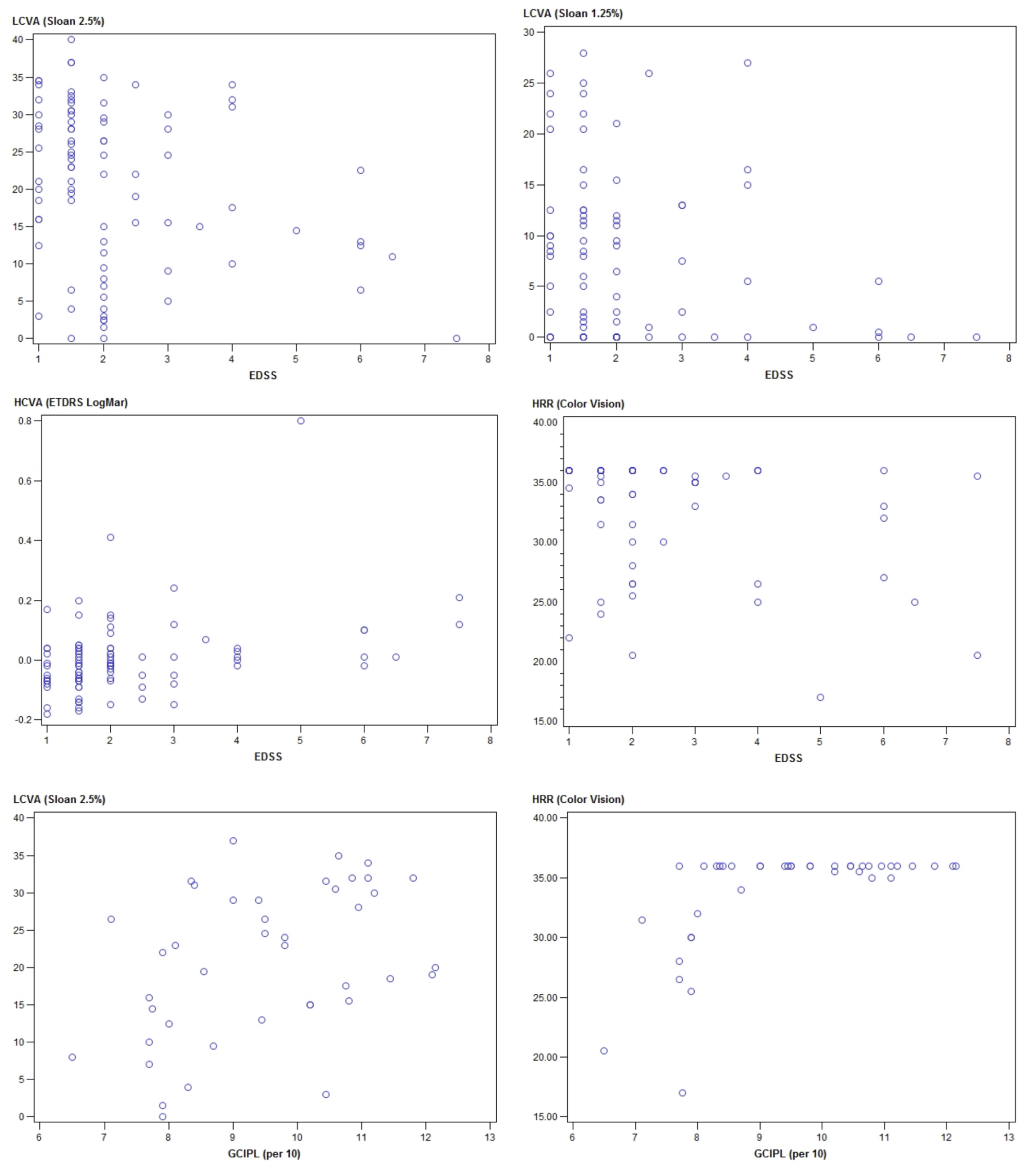
Correlation between EDSS or thickness of the GCIPL and visual acuity. The scatter-plots show the correlation between visual acuity measured with HCVA, LCVA (either 2.5% or 1.25% contrast) or color vision (HRR) with the EDSS. Moreover, the scatter-plot of the correlation of the visual acuity associated with the thickness of the GCIPL (the 2.5%LCVA and the HRR) is shown on the bottom panels.

As expected, MS patients with prior ON had significantly lower scores for LCVA and color vision than patients without a prior episode, whereas the differences between the subgroups for HCVA and quality of vision were not significant (**Tables 1 and S1**). The MS patients had a thinner retina, and the thickness of the pRNFL and the volume or thickness of the GCIPL were significantly lower in patients that had previously suffered ON than in patients that had not (**Fig 1**). Both measures of retina atrophy, pRNFL thickness and GCIPL volume, were significantly correlated with LCVA and color vision in the linear regression analyses adjusted for age, gender, history of ON, use of DMT and EDSS. The GCIPL volume showed a trend (p = 0.09) to be correlated with the combined score of the quality of vision (VFQ-25 +10 items) (**Table 2 and Fig 2**).

**Table 2:** Association between visual outcomes and retinal atrophy in patients with MS. Linear regression analyses adjusted by age, gender, history of ON, DMT use and EDSS. To analyze visual acuity, the retinal layer thickness was multiplied by 10 to readily visualize the values.

	**pRNFL (x10)**				**GCIPL (x10)**			
	**R**^**2**^ **(p-value)**	**Β**	**95% CI**	**p-value**	**R**^**2**^ **(p-value)**	**β**	**95% CI**	**p-value**
**HCVA**	0.2587 (0.0105)	-0.0002	-0.0004 / 0.00008	0.1932	0.3441 (0.0192)	-0.022	-0.0606 / 0.0161	0.2474
**2.5% LCVA**	0.3121 (0.0013)	0.032	0.0133 / 0.0506	0.0011	0.2809 (0.0656)	3.0	0.2764 / 5.7323	0.0319
**HRR**	0.4604 (p<0.0001)	0.016	0.0084 / 0.0235	<0.0001	0.4954 (0.0006)	2.1	1.0724 / 3.1110	0.0002
	**pRNFL**				**GCIPL**			
	**R**^**2**^ **(p-value)**	**Β**	**95% CI**	**p-value**	**R**^**2**^ **(p-value)**	**β**	**95% CI**	**p-value**
**VFQ-25**	0.1974 (0.0514)	0.139	-0.0963 / 0.3749	0.2411	0.1997 (0.2379)	29.8	-6.3531 / 66.0456	0.1030
**VFQ-25 +10 items**	0.1670 (0.1085)	0.116	-0.1097 / 0.3433	0.3060	0.1901 (0.2707)	29.6	-5.1002 / 64.3056	0.0921

### Cross-sectional predictive variables of visual outcomes

In order to identify which independent variables (predictors) might explain the visual outcomes, multiple regression analysis was performed to evaluate HCVA, LCVA, color vision and quality of vision. The independent variables included in all the models were sex, age, disease duration, previous ON, EDSS, MSFC, BRB-N z score, SDMT score, use of DMT, pRNFL thickness and GCIPL volume. The models analyzing the quality of vision also included HCVA, LCVA and color vision. The univariate analysis showed an association of HCVA with the EDSS, LCVA, HRR and the thickness of the pRNFL (**S2 Table**). The univariate analysis of the 2.5% LCVA found associations with EDSS, MSFC, DMT use, HCVA, HRR, thickness of the pRNFL and volume of the GCIPL (**S3 Table**). Regarding color vision, the variables associated in the univariate analysis were age, disease duration, previous ON, EDSS, HCVA, LCVA, pRNFL thickness and GCIPL volume (**S4 Table**). Finally, the variables associated with quality of vision were age, disease duration, EDSS, HCVA, LCVA, color vision and GCIPL volume (**S5 Table**).

The multivariate analysis for the HCVA showed that the only significant variable explaining HCVA was the EDSS (**Table 3**). In the case of LCVA (2.5% Sloan charts), the only significant variable in the stepwise approach was the GCIPL volume. Regarding color vision, the stepwise approach identified the GCIPL volume and the EDSS as explanatory variables. Finally, in terms of quality of vision, either VFQ-25 or the combined score with the 10 neuro-ophthalmological items, the explanatory variables were HCVA and color vision (**Table 3**).

**Table 3:** Multivariate linear regression analysis between the clinical variables and visual outcomes. The variables included are described in Table 1. The variables were selected using the stepwise method. To analyze visual acuity, the retinal layer thickness was multiplied by 10 to readily visualize the values.

	R^2^ (p-value)	variable	β	95% CI (upper / lower)	p-value
**HCVA**	0.1265 (0.0007)	EDSS	0.03074	0.01343 / 0.04806	0.0007
**2.5% LCVA**	0.1729 (0.0068)	GCIPL(x10)	2.8309	0.8257 / 4.8362	0.0068
**HRR**	0.4512 (<0.0001)	GCIPL(x10)	1.6885	0.94043 / 2.4366	<0.0001
		EDSS	-1.24477	-2.26885 / -0.22070	0.0186
**VFQ-25**	0.3431 (<0.0001)	HCVA	-26.3687	-46.2545 / -6.4830	0.0100
		HRR	0.8232	0.2308 / 1.4156	0.0071
**VFQ-25 + 10 items**	0.3537 (<0.0001)	HCVA	-26.1832	-44.7740 / -7.5924	0.0064
		HRR	0.7596	0.2058 / 1.3135	0.0078

The best model based on the R^2^ value obtained for visual acuity was that for color vision, which explained up to 45% of the HRR variability. Accordingly, a decrease of 1 unit in the GCIPL volume predicted a reduction of almost 1 point in the HRR and, a 1-point increase of the EDSS almost a 1-point decrease in the HRR. Regarding quality of vision, the models were similar for the VFQ-25 with and without the 10 neuro-ophthalmological items, and they explained almost 35% of the VFQ scores. As such, a 0.1 point increase in the HCVA predicted an almost 2.6 point decrease in the VFQ-25, whereas a 1 point decrease in the HRR predicted almost a 1 point decrease in the VFQ-25. Therefore, in our models, HCVA and color vision were the two variables that had significant effects on the quality of vision.

## Discussion

In this study we found impaired quality of vision was mainly dependent on HCVA and color vision. Color vision and LCVA are more sensitive test for detecting visual abnormalities for acute ON[6, 20], and as such, it was proposed to include such tests in MS patient assessments [2, 5, 7, 23]. However, our study identified that the main contributor to quality of vision was HVCA and color vision, probably due to the relevance of both types of visual function in the patient’s visual perception[2].

The multivariate analysis carried out here provides several insights into visual outcomes. HCVA was only dependent on global disability (EDSS) but not to retina atrophy, suggesting that this scale may be less sensitive to capture the damage observed in visual pathway in MS[2, 6]. In terms of LCVA and color vision, GCIPL volume was the main explanatory variable, confirming that the ganglion cell layer is the most sensitive retinal structure to explain the changes in visual function[24, 25]. As such, the GCIPL volume is proposed as a surrogate end-point to assess long-term visual disability[20, 25]. Indeed, the two best models to predict visual disability were those of color vision and quality of vision, although they explained not more than one third to a half of the variability. Accordingly, it is still necessary to develop accurate and sensitive measures of vision and predictive models that can be used to monitor patient disease course, and that can be used as accepted surrogate end-points in clinical trials.

Regarding quality of vision, we found that the main explanatory variables were HCVA and color vision but not LCVA or retinal atrophy. HCVA is critical for reading and other high definition vision functions; while changes in color vision are readily detected by patients and they alter their visual perception[2, 10]. Therefore, it may not be appropriate to use only retinal atrophy or LCVA as surrogates of quality of vision. Quality of vision is a complex outcome that involves not only visual acuity or color vision but also, motion perception, visual fields and other aspects related to the integration of overall visual activity in the patient’s daily activities. Like many other quality of life scales, individual assessment of visual sub-function is unlikely to explain its variability. In MS patients that have previously suffered ON and with a RNFL below 75μm, the VFQ-25 scores dropped approximately 2 points for every 1 μm decrease in the RNFL[26]. Moreover, quality of vision has been related to poor vision[27, 28], low contrast vision[7, 23] or contrast sensitivity[29]. Recently, quality of vision in MS patients was shown to be directly related with the thickness of the pRNFL (although it was not significant in Balk et al study (p = 0.06)) and the volume of GCIPL, both at the binocular and monocular level and irrespective of prior ON [25, 30]. However, in our study the multivariate analysis failed to confirm such associations, probably due to the low dispersion of the data in a mildly impaired population of MS patients compared with such studies, the use of a smaller sample size than Balk et al study or lack of power for stepwise analysis. Our finding that quality of vision was dependent on HCVA and color vision supports a close relationship between patient’s reported outcomes and neurological performance in the visual pathway.

There are several limitations to our study. First, we have performed a cross-sectional analysis, while longitudinal studies will be required to identify predictors of visual scores. Hence, we plan to perform a longitudinal assessment of the Barcelona MS-VisualPath cohort to validate our results. The cohort was of intermediate size and with a predominance of the RRMS sub-type, which limited our capacity to perform a stratified analysis based on disease duration or MS sub-type. Finally, a significant proportion of our cohort received DMTs and thus, our results are representative of current MS populations but they do not explain the natural history of the disease in terms of visual impairment.

In summary, our study shows that visual impairment is common in patients with MS and while it was more severe in patients that had previous suffered ON, it was also significant in patients that had not. The GCIPL volume was the most sensitive surrogate end-point, a parameter that can be used to monitor the disease course or as an end-point in clinical trials. Finally, we describe that HCVA and color vision are predictors of quality of vision.

## Supporting information

S1 Table 1.25% low contrast visual acuity assessment. (DOCX)

S2 Table Univariate association with high contrast visual acuity. (DOCX)

S3 Table Univariate association with low contrast visual acuity. (DOCX)

S4 Table Univariate association with color vision. (DOCX)

S5 Table Univariate association with vision-related quality of life. (DOCX)

S1 File Anonymized data set. (XLSX)

S2 File Raw data of the VFQ25 (Spanish version). (XLSX)
