# Supplementary material for: Predictors of vision impairment in Multiple Sclerosis
Source: PLoS One. 2018 Apr 17;13(4):e0195856. doi: 10.1371/journal.pone.0195856 (PMC5903642; doi:10.1371/journal.pone.0195856)
Supplement: S1 Table — (DOCX) [file pone.0195856.s001.docx]

**S1 Table. Results of the 1.25% low contrast visual acuity**

|  | | **No prior history of optic neuritis (N=65)** | **Prior history of optic neuritis (N=54)** | **Total (N=119)** |
| --- | --- | --- | --- | --- |
| LCVA (Sloan 1.25%) | n | 46 | 38 | 84 |
|  | N. missing | 19 | 16 | 35 |
|  | Mean (95%CI) | 9.95 (7.20 , 12.69) | 5.17 (3.12 , 7.22) | 7.79 (5.98 , 9.59) |
|  | Standard deviation (SD) | 9.24 | 6.24 | 8.33 |
|  | Median | 8.50 | 2.50 | 5.50 |
|  | (Q1, Q3) | (0.00, 16.50) | (0.00, 9.50) | (0.00, 12.50) |
|  | (Min, Max) | (0.00, 28.00) | (0.00, 25.00) | (0.00, 28.00) |
|  | P-value Wilcoxon |  |  | 0.0279 |
|  | | | | |
