# Supplementary material for: Predictors of vision impairment in Multiple Sclerosis
Source: PLoS One. 2018 Apr 17;13(4):e0195856. doi: 10.1371/journal.pone.0195856 (PMC5903642; doi:10.1371/journal.pone.0195856)
Supplement: S2 Table — (DOCX) [file pone.0195856.s002.docx]

**S2 Table. Univariate association with high contrast visual acuity (HCVA: ETDRS LogMar)**

| *Variable* | *Regression coefficients (b)* | *Standard Error* | *Lower 95% CL* | *Upper 95% CL* | *p-value* |
| --- | --- | --- | --- | --- | --- |
| Age (years) | 0.00166 | 0.00146 | -0.00125 | 0.00457 | 0.2590 |
| Gender | -0.02217 | 0.02979 | -0.08138 | 0.03704 | 0.4588 |
| Disease duration (years) | 0.00210 | 0.00191 | -0.00170 | 0.00589 | 0.2752 |
| MSFC Z score | -0.02634 | 0.01468 | -0.05566 | 0.00298 | 0.0774 |
| BRB Z Score | -0.02030 | 0.02393 | -0.07296 | 0.03236 | 0.4143 |
| SDMT | 0.00047273 | 0.00136 | -0.00248 | 0.00343 | 0.7334 |
| Use of DMD | 0.02380 | 0.03373 | -0.04326 | 0.09087 | 0.4823 |
| History of MSON | -0.05130 | 0.02709 | -0.10514 | 0.00254 | 0.0616 |
| EDSS | 0.03074 | 0.00871 | 0.01343 | 0.04806 | 0.0007 |
| LCVA (Sloan 2.5%) | -0.00588 | 0.00114 | -0.00815 | -0.00360 | **<.0001** |
| LCVA (Sloan 1.25%) | -0.00561 | 0.00165 | -0.00889 | -0.00233 | **0.0010** |
| HRR (Color Vision) | -0.01845 | 0.00243 | -0.02329 | -0.01361 | **<.0001** |
| pRNFL (per 10 µm) | -0.00026663 | 0.00009847 | -0.00046347 | -0.00006979 | 0.0087 |
| GCIPL (per 10 µm) | -0.02435 | 0.01560 | -0.05590 | 0.00719 | 0.1265 |
| Dependent variable: HCVA Univariate linear regression analyses |  |  |  |  |  |
