# Supplementary material for: Predictors of vision impairment in Multiple Sclerosis
Source: PLoS One. 2018 Apr 17;13(4):e0195856. doi: 10.1371/journal.pone.0195856 (PMC5903642; doi:10.1371/journal.pone.0195856)
Supplement: S3 Table — (DOCX) [file pone.0195856.s003.docx]

**S3 Table. Univariate association with low contrast visual acuity (LCVA: Sloan 2.5%))**

| *Variable* | *Regression coefficients (b)* | *Standard Error* | *Lower 95% CL* | *Upper 95% CL* | *p-value* |
| --- | --- | --- | --- | --- | --- |
| Age (years) | -0.16447 | 0.12175 | -0.40658 | 0.07764 | 0.1803 |
| Gender | 4.13397 | 2.51258 | -0.86087 | 9.12881 | 0.1036 |
| Disease duration (years) | -0.03757 | 0.16213 | -0.35999 | 0.28484 | 0.8173 |
| MSFC Z score | 2.67062 | 1.16035 | 0.35324 | 4.98800 | **0.0246** |
| BRB Z Score | 3.85496 | 3.11178 | -2.99402 | 10.70393 | 0.2412 |
| SDMT | 0.20923 | 0.17617 | -0.17461 | 0.59307 | 0.2579 |
| Use of DMD | -5.78219 | 2.77009 | -11.29278 | -0.27160 | **0.0400** |
| History of MSON | 1.59684 | 2.31695 | -3.00910 | 6.20279 | 0.4926 |
| EDSS | -2.14740 | 0.80283 | -3.74391 | -0.55088 | **0.0090** |
| HCVA (ETDRS LogMar) | -40.03648 | 7.78443 | -55.51141 | -24.56155 | **<.0001** |
| LCVA (Sloan 1.25%) | 0.98865 | 0.09257 | 0.80450 | 1.17281 | **<.0001** |
| HRR (Color Vision) | 1.21227 | 0.22246 | 0.76981 | 1.65472 | **<.0001** |
| pRNFL (per 10 µm) | 0.02649 | 0.00701 | 0.01249 | 0.04050 | **0.0004** |
| GCIPL (per 10 µm) | 2.83097 | 0.99138 | 0.82572 | 4.83622 | **0.0068** |
| Dependent variable: LCVA (Sloan 2.5%) Univariate linear regression analyses | | | | | |
