# Supplementary material for: Predictors of vision impairment in Multiple Sclerosis
Source: PLoS One. 2018 Apr 17;13(4):e0195856. doi: 10.1371/journal.pone.0195856 (PMC5903642; doi:10.1371/journal.pone.0195856)
Supplement: S4 Table — (DOCX) [file pone.0195856.s004.docx]

**S4 Table. Univariate association with color vision (HRR)**

| *Variable* | *Regression coefficients (b)* | *Standard Error* | *Lower 95% CL* | *Upper 95% CL* | *p-value* |
| --- | --- | --- | --- | --- | --- |
| Age (years) | -0.11524 | 0.05000 | -0.21468 | -0.01580 | **0.0237** |
| Gender | -1.08829 | 1.07122 | -3.21816 | 1.04157 | 0.3125 |
| Disease duration (years) | -0.13648 | 0.06529 | -0.26633 | -0.00663 | **0.0396** |
| MSFC Z score | 0.47331 | 0.48991 | -0.50569 | 1.45231 | 0.3377 |
| BRB Z Score | -0.29190 | 1.49085 | -3.57323 | 2.98944 | 0.8483 |
| SDMT | 0.03874 | 0.08288 | -0.14183 | 0.21931 | 0.6485 |
| Use of DMD | -1.20040 | 1.16922 | -3.52678 | 1.12599 | 0.3076 |
| History of MSON | 2.29672 | 0.93865 | 0.43044 | 4.16301 | **0.0165** |
| EDSS | -1.07905 | 0.30368 | -1.68304 | -0.47505 | **0.0006** |
| HCVA (ETDRS LogMar) | -21.85604 | 2.88374 | -27.58969 | -16.12240 | **<.0001** |
| LCVA (Sloan 2.5%) | 0.21737 | 0.03989 | 0.13803 | 0.29670 | **<.0001** |
| LCVA (Sloan 1.25%) | 0.20670 | 0.05701 | 0.09323 | 0.32018 | **0.0005** |
| pRNFL (per 10 µm) | 0.01580 | 0.00292 | 0.00997 | 0.02164 | **<.0001** |
| GCIPL (per 10 µm) | 1.80659 | 0.38972 | 1.01764 | 2.59553 | **<.0001** |
| Dependent variable: HRR Univariate linear regression analyses | | | | | |
