# Supplementary material for: Predictors of vision impairment in Multiple Sclerosis
Source: PLoS One. 2018 Apr 17;13(4):e0195856. doi: 10.1371/journal.pone.0195856 (PMC5903642; doi:10.1371/journal.pone.0195856)
Supplement: S5 Table — (DOCX) [file pone.0195856.s005.docx]

**S5 Table. Univariate association with vision-related quality of life (VFQ-25+10items)**

| *Variable* | *Regression coefficients (b)* | *Standard Error* | *Lower 95% CL* | *Upper 95% CL* | *p-value* |
| --- | --- | --- | --- | --- | --- |
| Age (years) | -0.28586 | 0.12500 | -0.53415 | -0.03757 | **0.0245** |
| Gender | 3.14503 | 2.30165 | -1.41871 | 7.70878 | 0.1747 |
| Disease duration (years) | -0.42573 | 0.15775 | -0.73908 | -0.11238 | **0.0083** |
| MSFC Z score | 1.85238 | 1.19169 | -0.52758 | 4.23235 | 0.1249 |
| BRB Z Score | -0.83499 | 4.28379 | -10.26354 | 8.59356 | 0.8490 |
| SDMT | 0.19051 | 0.23044 | -0.31158 | 0.69261 | 0.4245 |
| Use of DMD | -3.34848 | 2.92623 | -9.16662 | 2.46966 | 0.2557 |
| History of MSON | -0.10398 | 2.17840 | -4.42335 | 4.21539 | 0.9620 |
| EDSS | -2.45018 | 0.82396 | -4.08712 | -0.81325 | **0.0038** |
| HCVA (ETDRS LogMar) | -43.01496 | 7.76447 | -58.45547 | -27.57445 | **<.0001** |
| LCVA (Sloan 2.5%) | 0.34173 | 0.10665 | 0.12957 | 0.55389 | **0.0019** |
| LCVA (Sloan 1.25%) | 0.40191 | 0.14835 | 0.10657 | 0.69726 | **0.0083** |
| HRR (Color Vision) | 1.33422 | 0.23429 | 0.86805 | 1.80039 | **<.0001** |
| pRNFL | 0.15544 | 0.08806 | -0.02071 | 0.33158 | 0.0826 |
| GCIPL | 28.19510 | 12.94769 | 2.00593 | 54.38428 | **0.0355** |
| Dependent variable: NEI-VFQ-25 (Global Score) Univariate linear regression analyses | | | | | |
